# Kinetic Rate Constant Prediction Supports the Conformational Selection Mechanism of Protein Binding

**DOI:** 10.1371/journal.pcbi.1002351

**Published:** 2012-01-12

**Authors:** Iain H. Moal, Paul A. Bates

**Affiliations:** 1 Protein Interactions and Docking Laboratory, Life Sciences Department, Barcelona Supercomputing Center, Barcelona, Spain; 2 Biomolecular Modelling Laboratory, Cancer Research UK London Research Institute, London, United Kingdom; National Cancer Institute, United States of America and Tel Aviv University, Israel

## Abstract

The prediction of protein-protein kinetic rate constants provides a fundamental test of our understanding of molecular recognition, and will play an important role in the modeling of complex biological systems. In this paper, a feature selection and regression algorithm is applied to mine a large set of molecular descriptors and construct simple models for association and dissociation rate constants using empirical data. Using separate test data for validation, the predicted rate constants can be combined to calculate binding affinity with accuracy matching that of state of the art empirical free energy functions. The models show that the rate of association is linearly related to the proportion of unbound proteins in the bound conformational ensemble relative to the unbound conformational ensemble, indicating that the binding partners must adopt a geometry near to that of the bound prior to binding. Mirroring the conformational selection and population shift mechanism of protein binding, the models provide a strong separate line of evidence for the preponderance of this mechanism in protein-protein binding, complementing structural and theoretical studies.

## Author Summary

Almost all biological processes involve proteins interacting with each other. Knowledge about how quickly proteins associate and disassociate is fundamental for understanding how proteins work together to perform biological functions. Here we look at a large set of interacting protein pairs, which are extensively characterized by many numerical values that describe the properties of their interactions. An algorithm was used to automatically construct linear equations for the association and dissociation rates by selecting and weighting important features. Upon inspecting the selected features, we conclude that the most significant factor determining the rate of association is how often the unbound proteins can adopt the shape with which their surfaces complement each other. This suggests that proteins must adopt this configuration before they bind. Secondly, the rate at which proteins dissociate is determined by how strong the interaction is once this shape has been adopted, suggesting that proteins must dissociate before they adopt a more relaxed state. This work contradicts the view that proteins bind first and then adjust their shape, and instead supports the hypothesis that proteins adopt many shapes, and only those which are in the correct configuration are selected by their binding partner.

## Introduction

The rates at which biomolecules associate and disassociate are central to the behavior of biological systems and their determination is crucial to understanding and modeling how the systemic properties of networks evolve over time [1]–[5]. Thus, as research into the structural characterization of protein interaction networks advances [6]–[8], there is a growing need to construct accurate and efficient models for predicting kinetic rate constants; many systems cannot be understood only in terms of their equilibrium behavior. Constructing models of such networks using differential equations requires rate constants for all the relevant processes, and experimental values are frequently not available. For instance, TGF-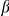 induced Smad signal transduction involves a dynamic network of processes, including phosphorylation, dephosphorylation, nucleocytoplasmic shuttling and complex formation [9]. Being able to estimate or measure as many rates as possible, and thus reducing the number of adjustable parameters, was imperative to building a quantitative model of predictive value. While little research has been performed on the process of biomolecular dissociation, the process of association is a topic of intense study. Much work has focused on the diffusion-limited association of reactive surfaces and the role of long-range steering forces, transitions states and encounter complexes [10], [11]. Rigid-body Brownian dynamics has proven to be a highly effective and popular tool for the simulation of association trajectories. However, the role of flexibility has been largely neglected due to the complexity it engenders. A very different approach to modeling kinetic rates is taken here. Instead of simulating the association process itself, or characterizing the energy landscape, a feature selection algorithm is applied to infer rate constants from structural and energetic properties derived from the structures of complexes and their unbound constituents. To avoid overfitting, models are selected using a form of regularization, in which each pair of 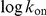 and 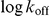 models are combined to form a 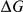 binding free energy function. The pair of rate constant models best able to predict the binding free energy of a separate set of interactions is selected. These models are then validated using a third set of binding free energy data. A large set of binding affinities is used [12], with various model training, selection and validation sets delineated according to the overall quality of the data, as previously determined by the extent to which the affinities have been experimentally characterized [13]. As empirical rate constants are neither required for model selection nor validation, all the complexes for which kinetic data are available can be used for training.

A number of binding mechanisms have been proposed. The earliest is the lock-and-key model, in which molecules bind rigidly with pre-organized complementarity [14]. This was followed by the induced fit model, in which molecules bind in an unbound conformational state, with the bound state induced by the field provided by the binding partner [15]. A more recently proposed mechanism is the conformational selection model, in which the bound state lies within the pre-existing equilibrium of the unbound molecule and is sequestered by the binding partner, thus shifting the equilibrium toward this state [16], [17]. This mechanism has since been expanded to include scenarios in which certain conformations are selected followed by induction to the final structure [18]. For protein-protein interactions, structural studies including normal mode analysis, crystallography and nuclear magnetic resonance, have shown the presence of conformations similar to the bound within the accessible ensemble of unbound molecules [18]–[20], supporting the conformational selection model. However, excursions into the bound state do not necessarily imply conformational selection [21], and in order to demonstrate that this mechanism is indeed followed, it is necessary to show that interactions occur only with the small subpopulation of the molecules which are organized to complement their binding partner. One way of showing this is to empirically demonstrate, on a diverse set of complexes, the distinctive kinetics which distinguishes conformational selection from other binding mechanisms [22].

### Previous Work

Previously, we complied a benchmark of 144 protein-protein binding affinities from the literature, for which bound and unbound structures are available [12]. For these, we calculated a set of 200 molecular descriptors describing various aspects of the interaction and the observed conformational changes [13]. Although some descriptors relate to the composition or geometry of the interface, most were derived from energetic models. These include Coulombic and continuum electrostatics models, hydrophobic burial and Van der Waals terms, as well as four-body and two-body statistical potentials. Other potentials were included to model 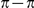, cation-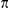, H-bond and aliphatic interactions. Also included were models of translational, rotational, vibrational, side chain and disorder to order transition entropy changes. Many of the descriptors were also averaged over structural ensembles derived using the CONCOORD package [23]. Although the complete descriptor set was fully described previously [13], details of those which are highlighted in the current work are shown in Table 1. As a significant number of incorrect kinetic rates and binding affinities are reported in the literature, often due to methodological limitations and sometimes differing by several orders of magnitude for the same complex, we assembled a subset of the affinity benchmark for which high confidence could ascribed to the reported affinities. For this validated set of interactions, similar affinities were independently determined by more than one group or biophysical technique [13]. All the experimental sources used to construct this validated set, and a detailed summary of their methods and conditions, can be found at the website for affinity benchmark (http://bmm.cancerresearchuk.org/%7Ebmmadmin/Affinity/). More detailed discussions regarding the experimental data and the construction of the validated set can be found elsewhere [12], [13].

**Table 1:** Molecular descriptors.

Term	Description
DFIRE	The DFire atomistic distance potential [26], [47]
OPUS_PSP	The OPUS-PSP orientational atomistic contact potential [48]
OPUS_CA	The OPUS-CA combined residue level potential [49]
DDFIRE	The DDFire orientational atomistic distance potential [50]
ATOM_P	The proportion of polar atoms at the interface [51]
RES_C	The proportion of charged residues at the interface [51]
QP_PP	The REFINER residue level contact potential [52],see [53]
MJPL_PP	The residue level contact potential reported in [54], see [53]
RO_PP	The residue level contact potential reported in [55], see [53]
MJ2H_PP	The residue level contact potential reported in [56], see [53]
GEN_4_BODY	A four-body residue level contact potential [53], [57]
SASA	The SASA solvation model [58], as implemented in CHARMM [59]
LK_SOLV	The EEF1 solvation model [60], as implemented in CHARMM [59]
NUM_HB	The number of interfacial hydrogen bonds [51]
H_BOND	The hydrogen bonding potential implemented in FireDock [61]
ROS_HBOND	The hydrogen bonding potential implemented in PyRosetta [62]
ROS_FA_ATR	The London dispersion energy implemented in PyRosetta [62]
ROS_CG	The PyRosetta coarse-grain potential [62]
ROS_CG_BETA	The PyRosetta coarse-grain C 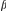 potential [62]
ROS_CG_VDW	The PyRosetta coarse-grain Van der Waals potential [62]
NIP	An interface packing score [63]
STC_H	A simple binding enthalpy score [64]
STC_S_SC	A side-chain entropy model [64]
S_WLC_INT2	A disorder to order transition entropy model [65]

Descriptions of the basic molecular descriptors highlighted in this work. Where descriptors appear in the text without suffix, this indicated that values are either computed directly or as changes upon complexation, calculated as the difference between the bound complex and the unbound protein in the bound conformation. Those appearing suffixed with _UB pertain to the conformational changes upon binding, and are calculated as the difference between unbound proteins in the bound and unbound conformations. The suffixes _ENS and _EBU respectively correspond the interaction and conformation descriptors which are averaged over conformational ensembles. Briefly, CONCOORD 2.1 was used to generate 100 conformations surrounding the complex and its unbound constituents [23]. Descriptors are calculated using mean values derived from these ensembles.

### Approach

**Figure 1:**
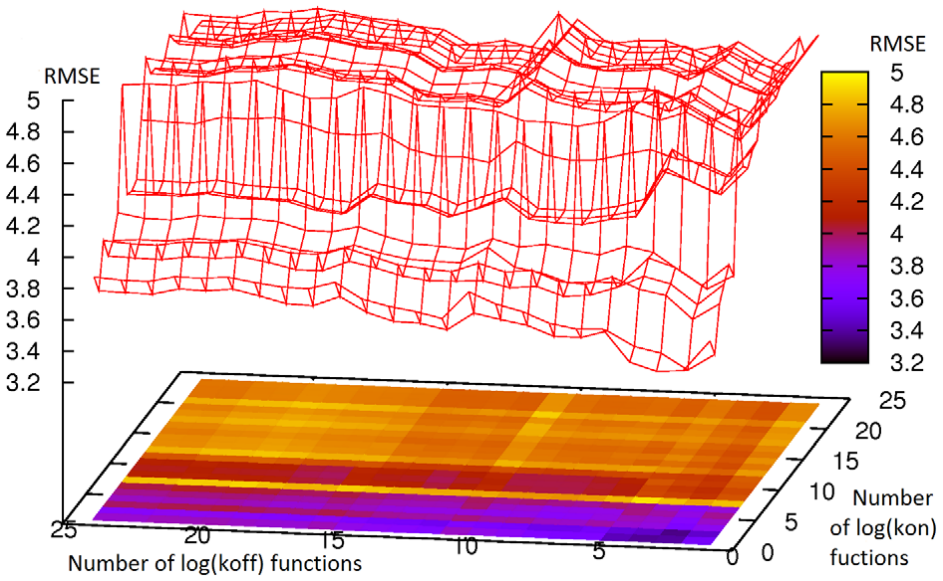
An early stopping surface. The surface shows how the RMSE of the predicted binding free energies of the test set, calculated via equation 1, vary with the number of features used in the rate constant models. This surface correspond to scheme 2 in Table 4. The 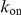 and 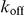 models which are selected, which use two features each, corresponds to the RMSE minimum.

In order to test the ability to infer kinetic rates from structural properties, interactions with empirical rate constants must be found for which unbound and bound structures exist. First, a benchmark of rate constants was derived from data in the literature. Of the 144 complexes in the affinity benchmark, association and dissociation rates could be found for 44, of which 27 are in the intersection with the set of affinities which have been determined by multiple experiments, and are thus known with high confidence. As this is a small number of data points, it is undesirable to divide them into separate sets for training, model selection and validation. However, the fundamental relationship between binding affinity and kinetics, given in equation 1, allows the predictive value of a pair of rate constant models to be evaluated on interactions for which binding affinities are available.
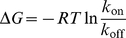
(1)This allows us to perform model selection using a variation of early stopping regularization [24]. In its original form, data is separated into a training and a test set. A greedy algorithm is used to iteratively train a predictive model. Initially, as the model is refined, its performance improves when evaluated on both the test and training data. However, as the model starts to overfit the training data, its performance on the test set diminishes whilst continuing to improve when evaluated on the training set. The model of greatest predictive value, which corresponds to the stationary point on the early stopping curve, is selected. Usually a third data set is required to obtain a good estimate of the generalization error. In the work presented here, the early stopping curve is replaced by an early stopping surface. An iterative feature selection and regression algorithm is used to produce a series of rate constant models. Each combination of 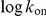 and 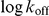 model is combined using equation 1 to produce a binding free energy model, which is evaluated on a test set of affinities to produce an early stopping surface. The stationary point on this surface is then used for model selection. An example of an early stopping surface is given in Figure 1. Finally, the ability of this pair of models to predict binding free energy is evaluated using a separate set of validation data, which has not been seen by either the training or selection process.

While a similar approach has been undertaken previously [25], model training, feature selection and model evaluation was performed on the same set of interactions, rendering the models highly susceptible to overfitting. Although leave-one-out cross-validation was employed, this was at the final evaluation stage and not as an outer wrapper. Further, as redundancy was not accounted for, and homologous pairs existed within the data set, the reported performance is susceptible to repeat example bias. Attempts to reconstruct these models failed to reproduce the correlations between predicted and experimental rate constants and affinities when applied to the rate constant data presented here, or the binding affinity benchmark and set of high-confidence affinities described previously [12], [13]. By clearly separating training, selection and validation sets, and controlling for repeat example bias, these potential sources of bias are eliminated in the presented work.

## Results

### Empirical Rate Constants

Kinetic rate constants for 44 complexes were compiled from the literature, and can be found in the supplementary information (Table S1). These complexes span a range of affinities from tens of femtomolar to micromolar, with 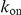 ranging from 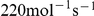 to 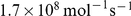 and 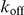 ranging from 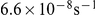 to 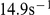. They also undergo a range of conformational changes, with interface RMSD changes ranging from 0.28 Å to 3.79 Å. These complexes have a wide variety of functions, with 18 complexes involving enzymes (14 interacting with inhibitors, 2 with substrates and 2 other interactions), 10 antibody/antigen complexes, 8 complexes with receptors and 8 other miscellaneous interactions of various function. The empirical on rates and off rates, along with their corresponding molecular descriptor sets, can be found in the supplementary information (Dataset S1 and Dataset S2).

As a preliminary investigation, we checked for correlations between the molecular descriptors and the rates. Standard significance of correlation tests was used to identify relevant descriptors. As this test was employed to find significant correlations, as opposed to evaluating single hypotheses, a strict criteria of 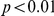 was used (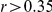 for 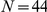). Although no such correlations were found with the 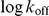 values, a number of significant correlations could be found for 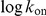, as shown in Table 2. Most notably are five correlations with energetic terms associated with the unbound to bound conformational change, one of which is a H-bonding energy (ROS_HBOND_UB) and the remainder of which are averaged over structural ensembles. Three of these are all-atom statistical pair potentials (DFIRE_EBU, OPUS_PSP_EBU and DDFIRE_EBU), and the other is a coarse-grained pair potential (OPUS_CA_EBU). The remaining significant correlations are with one of the H-bonding potentials calculated over the interface and averaged over structural ensembles (H_BOND_ENS), the number of hydrogen bonds across the interface (NUM_HB) and the proportion of interface atoms that are polar (ATOM_P). When repeated using only the rates for the intersection with the validated set (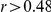 for 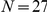), again no highly significant correlations could be found with 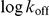, however a greater number of significant correlations could be found with 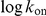, as shown in Table 3. As well as changes in conformational energy upon binding, calculated with atomistic pair potentials and averaged over conformational ensembles (DFIRE_EBU and OPUS_PSP_EBU), were all of the terms relating to intermolecular hydrogen bonding in the descriptor set (H_BOND, H_BOND_ENS, ROS_HBOND, ROS_HBOND_ENS and NUM_HB), a number of coarse-grained statistical pair potentials, calculated across the interface (QP_PP, MJPL_PP and RO_PP), two London dispersion energy terms (ROS_FA_ATR and ROS_FA_ATR_ENS), a side chain entropy term (STC_S_SC_ENS) and desolvation terms calculated using continuum electrostatics models (SASA, LK_SOLV and LK_SOLV_ENS).

**Table 2:** Significant correlations between association rates and molecular descriptors.

Descriptor	Correlation
DFIRE_EBU	−0.47
OPUS_PSP_EBU	−0.40
OPUS_CA_EBU	−0.40
DDFIRE_EBU	−0.38
H_BOND_ENS	−0.35
ROS_HBOND_UB	−0.35
ATOM_P	0.39
NUM_HB	0.39

Significant (p<0.01) correlations between association rates and molecular descriptors using the 44 complexes for which kinetic data is available.

**Table 3:** Significant correlations between association rates and molecular descriptors for the validated set.

Descriptor	Correlation
OPUS_PSP_EBU	−0.60
H_BOND_ENS	−0.59
ROS_HBOND_ENS	−0.56
H_BOND	−0.56
DFIRE_EBU	−0.56
QP_PP	−0.52
ROS_FA_ATR_ENS	−0.49
ROS_HBOND	−0.49
STC_S_SC_ENS	−0.48
MJPL_PP	−0.48
ROS_FA_ATR	−0.48
SASA	0.48
LK_SOLV	0.49
LK_SOLV_ENS	0.51
RO_PP	0.52
NUM_HB	0.57

Significant (p<0.01) correlations between association rates and molecular descriptors using the 27 complexes for which kinetic data is available and the binding affinity is known with high confidence.

### Model Training, Selection and Validation

**Figure 2:**
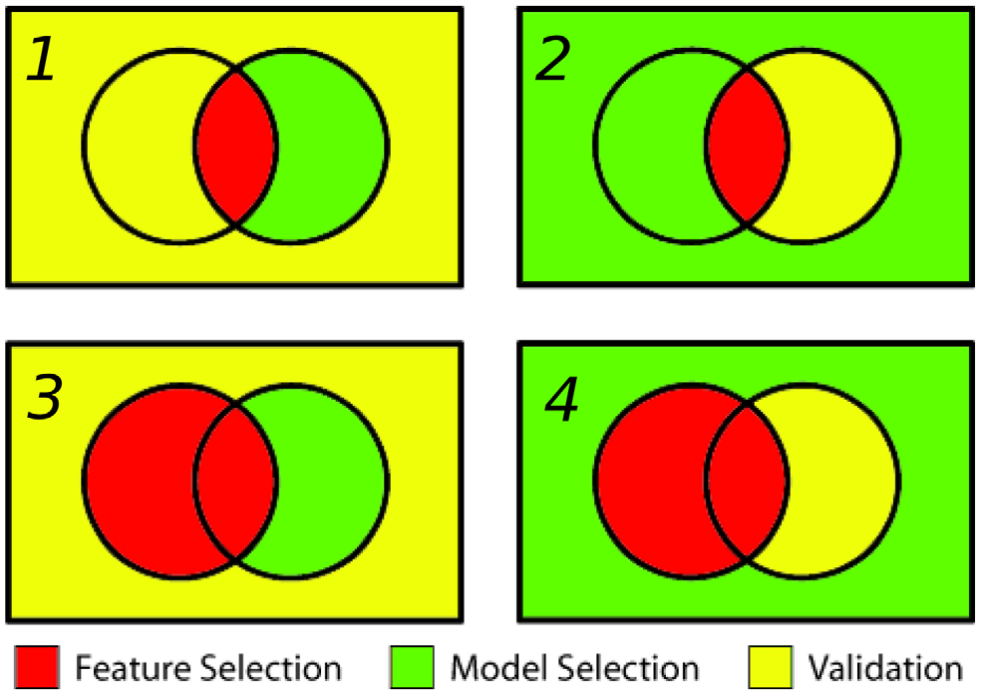
A Venn Diagram showing the four combinations of training, model selection and validation sets. Rectangles corresponds to all 137 complexes in the binding affinity benchmark [12]. The left circle corresponds to the 44 complexes for which kinetic data could be found. The right circle corresponds to the set of 57 complexes with high confidence affinities. These are the complexes for which similar affinities have been determined in multiple experimental setups, as previously determined [13]. The intersection of these sets contains 27 complexes.

**Figure 3:**
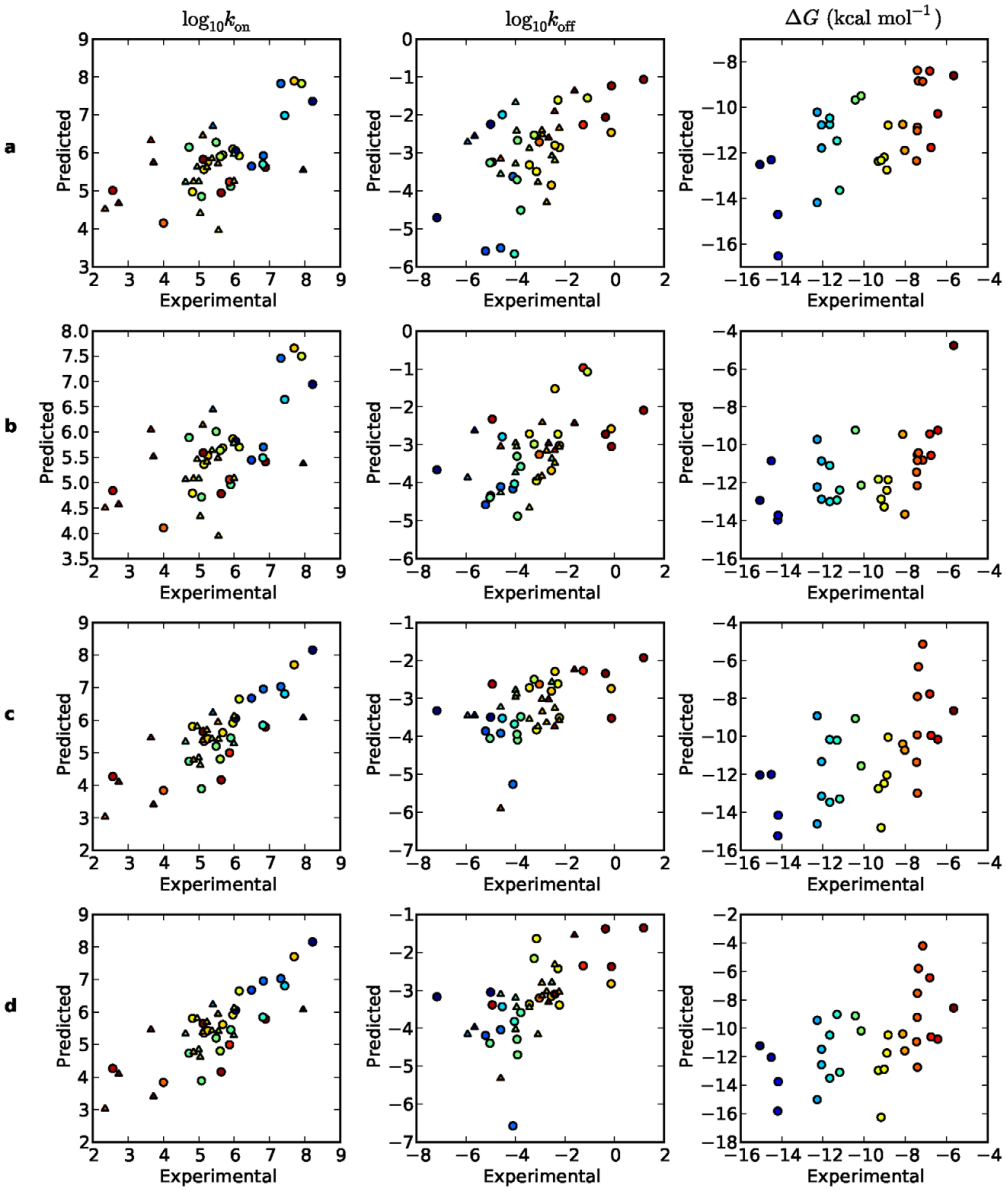
Models a, b, c and d. The 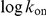 and 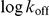 models, applied to the all the complexes for which kinetic data is available (with outlier 2OZA omitted from models **c** and **d**). Complexes in the intersection with the high confidence interactions are shown as circles, with the remainder shown as triangles. Points are coloured according to binding affinity. The combined 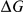 predictions, applied to the validation set, are also shown. These correspond to the set of high confidence affinities for which the rate constants are not known.

A number of considerations needed to be made in the preparation and implementation of the training, selection and validation scheme. These include whether or not to include outliers, choosing a performance metric for model selection, choosing between data quality and data quantity for model training, and whether high quality data should be preferentially allocated for model selection or model validation. As there are no hard and fast rules for making such decisions, the process was repeated a number of times with different configurations. Firstly, the binding affinity benchmark was partitioned into training, selection and validation sets in four ways, as shown in Figure 2. For model selection, two performance metrics were tested: the Pearson product-moment correlation coefficient (henceforth referred to simply as correlation) and the root mean square error (RMSE). Finally, the process was repeated both with and without the p36 MAPK/MK2 interaction (pdb 2OZA), which has an anomalously large binding interface and, upon binding, undergoes two large disorder to order transitions, in a loop and at the C-terminal region. The results for these runs can be seen in Table 4 and Table 5. The molecular descriptors that were selected for each model, and their weights, can be found in the supplementary information (Table S2). The four pairs of rate constant models which perform the best were selected for further analysis. (**a**) Scheme 2, selecting by RMSE, with outlier included. (**b**) Scheme 4, selecting by RMSE, with outlier included. (**c**) Scheme 4, selecting by RMSE, with outlier omitted. (**d**) Scheme 4, selecting by correlation, with outlier omitted. The functional form of these models and their performance are shown in Table 6. Scatter plots comparing the predicted and experimental 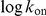 and 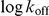 values for these models are shown in Figure 3, along with their final predictions when combined and applied to the complexes in the validation set. Of the best performing models shown in Table 6, a number of commonalities are observed. For **a** and **b**, the same 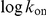 model was selected, consisting of two terms. The first of these is the number of intermolecular hydrogen bonds (NUM_HB) and the second is the energy change associated with the conformational changes that occur upon binding. These are averaged over conformational ensembles and are calculated using an atomistic pair potential (DFIRE_EBU). Methods **c** and **d** also selected the same association rate model. This consists of 7 descriptors which, in addition to NUM_HB and DFIRE_EBU, contains the proportion of interfacial residues that are charged (RES_C), a course-grained Van der Waals potential (ROS_CG_VDW), a simple binding enthalpy estimate (STC_H), the conformational energy change as calculated with a coarse-grained four-body statistical potential (GEN_4_BODY_UB), and an estimate of the entropy changes of interfacial loops which undergo a disorder to order transition (S_WLC_INT2). For the protein dissociation rate functions, **a**, **b**, **c** and **d** all selected different models. In **a**, two terms were selected, both interaction energies calculated using coarse-grain pair potentials, one a C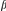 potential (ROS_CG_BETA), and the other a C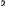 potential averaged over structural ensembles (OPUS_CA_ENS). For **b**, ROS_CG_BETA was selected, as was an interface packing score (NIP). For **c**, a single term was selected, a coarse-grained interaction potential (MJ2H_PP), while for **d** the MJ2H_PP potential was selected alongside MJPL_PP_UB, the conformational energy change as calculated with a coarse-grained potential.

**Table 4:** Results for training, model selection and validation.

		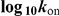			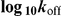			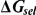		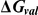		
Sel.	Scheme	#	Corr.	RMSE	#	Corr.	RMSE	RMSE	Corr.	RMSE	Corr.	p
RMSE	1	2	0.70	0.89	5	0.79	1.17	2.45	0.69	3.59	0.09	0.45
	2	2	0.70	0.89	2	0.56	1.58	3.36	0.10	2.61	0.59	**<0.01**
	3	8	0.77	0.86	2	0.45	1.47	2.50	0.60	3.67	0.19	0.14
	4	2	0.53	1.14	2	0.45	1.47	3.26	0.17	2.80	0.51	**<0.01**
Corr.	1	2	0.70	0.89	6	0.82	1.10	2.54	0.69	3.54	0.12	0.29
	2	5	0.83	0.69	4	0.72	1.31	3.94	0.22	3.27	0.39	**0.03**
	3	3	0.61	1.06	18	0.90	0.73	2.80	0.72	3.84	0.03	0.85
	4	10	0.80	0.80	2	0.45	1.47	3.67	0.27	2.87	0.43	**0.02**

Results for feature selection, model selection and validation, using the two selection criteria and the four data partitioning schemes. The number of features for the 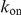 and 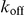 models is shown (#), alongside their leave-one-out cross-validation correlations and RMSE. The RMSE and correlation of the 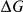 values used for selecting these models is also shown, as are those when the model is applied to the validation set, along with the significance of correlation.

**Table 5:** Results for training, model selection and validation (2OZA omitted).

		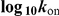			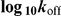			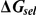		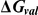		
Sel.	Scheme	#	Corr.	RMSE	#	Corr.	RMSE	RMSE	Corr.	RMSE	Corr.	p
RMSE	1	1	0.48	1.06	4	0.80	1.15	2.84	0.51	3.76	0.08	0.48
	2	1	0.48	1.06	2	0.58	1.54	3.66	0.00	2.91	0.48	**<0.01**
	3	9	0.80	0.78	5	0.73	1.11	2.36	0.72	3.46	0.25	**0.05**
	4	7	0.72	0.91	1	0.38	1.51	3.16	0.32	2.66	0.59	**<0.01**
Corr.	1	1	0.48	1.06	5	0.85	1.01	2.95	0.52	3.94	0.09	0.43
	2	2	0.65	0.92	21	1.00	0.00	4.12	0.31	3.86	0.39	**0.03**
	3	9	0.80	0.78	5	0.73	1.11	2.36	0.72	3.46	0.25	**0.05**
	4	7	0.72	0.91	2	0.51	1.43	3.18	0.33	2.55	0.60	**<0.01**

Results for feature selection, model selection and validation, using the two selection criteria and the four data partitioning schemes. The outlier, 2OZA, was omitted from these runs. The number of features for the 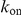 and 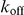 models is shown (#), alongside their leave-one-out cross-validation correlations and RMSE. The RMSE and correlation of the 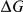 values used for selecting these models is also shown, as are those when the model is applied to the validation set, along with the significance of correlation.

**Table 6:** Selected models.

	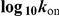			Error		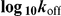			Error	
	Feat.	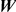	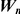	RMS	RMS 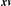	Feat.	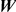	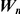	RMS	RMS 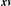
**a**	CONSTANT	4.29	-	0.81	0.89	CONSTANT	−2.11	-	1.41	1.58
	NUM_HB	7.29e-2	0.52			ROS_CG_BETA	−6.77e-1	−0.73		
	DFIRE_EBU	−3.60e-3	−0.50			OPUS_CA_ENS	3.77e-2	0.67		
**b**	CONSTANT	4.18	-	1.05	1.14	CONSTANT	−6.32	-	1.39	1.47
	NUM_HB	7.09e-2	0.39			ROS_CG_BETA	−4.89e-1	−0.52		
	DFIRE_EBU	−3.19e-3	−0.47			NIP	8.61e3	0.51		
**c**	CONSTANT	5.80	-	0.76	0.90	CONSTANT	−0.87	-	1.44	1.52
	RES_C	−6.87e-2	−0.53			MJ2H_PP	1.20e-2	0.46		
	NUM_HB	7.99e-2	0.42							
	ROS_CG_VDW	−1.01	−0.27							
	STC_H	−5.84e-2	−0.28							
	GEN_4_BODY_UB	1.43e-2	0.39							
	DFIRE_EBU	−2.76e-3	−0.41							
	S_WLC_INT2	−2.77e-1	−0.19							
**d**	CONSTANT	5.80	-	0.76	0.90	CONSTANT	−0.67	-	1.29	1.43
	RES_C	−6.87e-2	−0.53			MJ2H_PP	1.36e-2	0.53		
	NUM_HB	7.99e-2	0.42			MJPL_PP_UB	3.98e-3	0.40		
	ROS_CG_VDW	−1.01	−0.27							
	STC_H	−5.84e-2	−0.28							
	GEN_4_BODY_UB	1.43e-2	0.39							
	DFIRE_EBU	−2.76e-3	−0.41							
	S_WLC_INT2	−2.77e-1	−0.19							

The four models which were selected for further analysis. For each feature, absolute weights (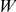) and normalized weights (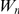), found after converting to z-scores, are shown. The term CONSTANT refers to the constant determined during regression. Root mean square error (RMS) and leave-one-out cross-validated error (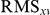) are also shown.

## Discussion

Not all of the runs shown in Table 4 and Table 5 produced models of good predictive value, and on occasions models with an inordinate number of adjustable parameters are selected, including one 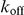 model with almost as many parameters as examples and with a leave-one-out cross-validated correlation differing from unity only at the 7th decimal place. Although such instabilities are inevitable when learning and selecting with such a small data set, most of the runs did produce models of reasonable size and predictive value. For comparison with other methods, the affinity of the complexes in the various subsets used for selection and validation was also calculated using the potentials of mean force described by Liu et al. [26] and Su et al. [27]. Calculated for the relative complement of the interactions with kinetic data in the validated set, which corresponds to the complexes used for selection in schemes 1 and 3 and for validation in schemes 2 and 4, these methods reproduced the affinities with a correlation of 0.59 and 0.62 respectively, and with RMSEs of 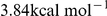 and 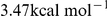. For the complement of the validated set, which is used for validation in scheme 1 and selection is scheme 2, the potentials achieve a respective correlations of 0.25 and 0.21, and RMSEs of 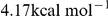 and 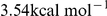. When evaluated on the complement of the unison of the validated set and the set of complexes with kinetic data, which is used for validation in scheme 3 and selection in scheme 4, the potentials of mean force predict the affinities with a correlations of 0.33 and 0.29 respectively, and with RMSEs of 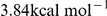 and 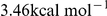.

Compared to the runs where the outlier is omitted (Table 5), both schemes 1 and 3 select models of lower RMSE than the potentials of mean force, irrespective of whether RMSE or correlation is chosen as the criterion for model selection (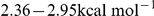 versus 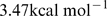 and 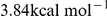). Although a pair of models with lower correlation is chosen in scheme 1, which fails to generate a significant correlation when validated, scheme 3 generated a pair of models which also outperforms both potentials in terms of correlation (0.72 versus 0.62 and 0.59). This pair of models, with 9 terms for 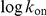 and 7 terms for 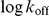, performs favorably compared to the potentials of mean force in terms of RMSE (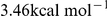, versus 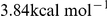 and 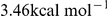), although it performs slightly worse in terms of correlation (0.25 versus 0.33 and 0.29). In scheme 2, and when correlation is used for model selection, the pair of models has poor RMSE, and when RMSE is used for selection, the models have poor correlation. Subsequently, when validated, these models fare poorly when compared to the potentials of mean force and with scheme 4. Indeed, the poor performance of schemes 1 and 2 compared to 3 and 4, suggests that the inclusion of extra data outside of the validated set for model training and feature selection improves the quality of the generated models. Generating, selecting and validating with scheme 4 produced the best models. On the selection set, this scheme performs comparably to the potentials of mean force in terms of correlation (0.32 and 0.33 versus 0.33 and 0.29) and is superior in terms of RMSE (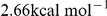 and 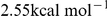, versus 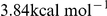 and 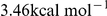). Similarly, when evaluated on the validation set, comparable performance is obtained in terms of correlation (0.59 and 0.60 versus 0.59 and 0.62), and an improved performance in terms of RMSE (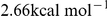 and 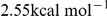 versus 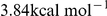 and 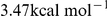). Overall, similar trends are seen when the outlier is included (Table 4). However, the best performing model is generated using scheme 2 and with RMSE as the selection criterion. The rate constant models are very simple, with only 2 features each. Despite a poor correlation with the model selection set (0.10), the model performs well on the high quality validation set, with a correlation of 0.59 and an RMSE of 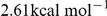, compared to 0.59 and 0.62, and 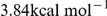 and 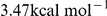 for the potentials of mean force.

### Implications for Association Rate

The strong linear relationship between the association rate constant and the energy difference between the unbound and bound conformational states, shown in most of the selected 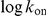 models, including **a**, **b**, **c** and **d**, is highly indicative. The relative number of unbound proteins in the bound conformational state compared to the unbound conformation is given by the equilibrium constant for the two states, equal to the ratio of their Boltzmann factors
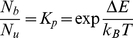
(2)The 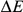 term corresponds to the energy difference between the bound and the unbound conformational ensembles. This is modelled here with the DFIRE_EBU descriptor, in which the mean energy of bound and unbound structural ensembles are calculated using the DFIRE statistical pair potential [26]. The inclusion of this term in the 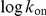 model has a clear physical interpretation; the rate of association depends linearly on the proportion of unbound proteins in the bound conformational ensemble. This mirrors exactly the conformational selection and population shift mechanism of protein binding. For instance, in the kinetic rate model of the conformational selection regime proposed by Weikl and von Deuster [22], association is dominated by the process
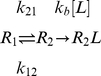
(3)For which the composite rate constant can be related to the pre-equilibrium constant as
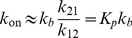
(4)


It has been noted that the highest affinity complexes tend to undergo only small conformational changes upon binding, although there are many exceptions. These observations can be explained by the energetic penalty associated with adopting a conformation far from the native. In light of the conformational selection model, these effects should be visible in the association rate constants. For instance, the interaction between the chemotaxis proteins CheY and CheA (1FFW), which undergoes significant changes at the binding interface (IRMSD 1.43 Å) has low binding affinity (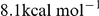 [28]), due to slow association kinetics (around 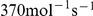 [28]). Conversely, the Acetylcholinesterase/Fasciculin interaction (1MAH), involves little structural rearrangement (IRMSD 0.61 Å), is strong (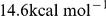 [29]) and undergoes fast association (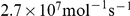 [30]). However, some complexes do not fit this pattern. For instance, the complex between Fab 44.1 and HEW lysozyme (1MLC) undergoes only minor conformational change (IRMSD 0.60 Å), yet has a small rate of association (around 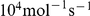 [31]). Similarly, the Erythropoietin/EPO receptor complex undergoes large conformational changes (IRMSD 2.44 Å), yet associates quickly (around 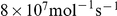 [32]). When the energetics of the respective conformational changes are taken into consideration the discrepancy disappears; the difference in mean energy between the bound and unbound ensembles, as indicated by the DFIRE_EBU descriptor, shows that the energy of the bound conformational ensemble of Fab 44.1/HEWL, relative to the unbound, is approximately 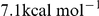 higher than for the EPO/EPOR complex. Thus, the bound ensemble of EPO/EPOR is more frequently visited in solution than those of Fab 44.1/HEWL, despite the greater extent of conformational change compared to the bound.

In the induced fit regime, however, the association follows the process
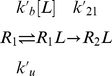
(5)From this, it can be shown that 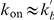 [22], and thus the rate of association is limited by the rate of diffusional encounter complex formation of the proteins in their unbound conformational ensemble. Hence the correlation and predictive value of the 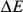 term shown here cannot be rationalizsed in the induced fit regime. In the conformational selection regime, models **a** and **b** suggest that hydrogen bonding is one of the strongest determinants of 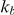, the association rate for proteins already in the bound conformation. Models **c** and **d** also have a hydrogen bonding term with a large normalized coefficient, as well as a highly weighted term reflecting the proportion of interface residues that are charged. The role of charged interfacial residues is of little surprise, as the ability of electrostatic steering forces to module protein association rates via long-range ionic interactions is well known [10], [11]. Perhaps more surprising is the prominence of hydrogen bonding. Although hydrogen bonds are also electrostatic in nature, the forces of charge-dipole and dipole-dipole interactions attenuate as 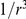 and 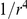 respectively and become negligible with increased separation. Further, the descriptor set contains solute-solute electrostatics terms and changes in solvent-solute electrostatics as calculated using a number of continuum models. However, NUM_HB is selected over these terms during model training, using both training sets, and models containing this term were selected during model selection by both of the disjoint model selection sets. Thus the role of hydrogen bonding cannot be explained by general electrostatic phenomenon such as electrostatic steering. A better explanation is the influence of solvent structure during the incipient interaction [33], [34]. Long-range order in liquid water, mediated by hydrogen bonds, allows correlation of molecular orientations on the scale of tens of nanometers [35], and may provides a means of intermolecular communication [36]. Indeed, long-range water-mediated hydrogen bonding has been implicated as an important stabilizing factor for protein folding intermediates [37]–[39]. Recently, in a molecular dynamics study of barase/barstar association, the non-contacting binding partners were stabilized by the solvent bonding network, and the restructuring of the solvent resulted in a reduced dielectric and enhanced electrostatics [34]. The number of interfacial hydrogen bonds may be indicative of the potential for such solvent mediated hydrogen bonding networks to form, enhance electrostatics, and stabilize the intermediates in the association pathway. If so, the results presented here suggest that these effects are important determinants of protein association rates in a wide range of protein-protein complexes, and that they can be experimentally probed via association kinetics.

### Implications for Dissociation Rate

Interestingly, with the sole exception of MJPL_PP_UB, all of the terms in the dissociation rate functions for models **a**, **b**, **c** and **d** relate to the interaction, and not to the energetic changes associated with the unbound to bound transition. In the conformational selection kinetic scheme proposed by Weikl and von Deuster [22], unbinding is dominated by the process
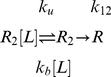
(6)Conformational relaxation usually occurs on the timescale of picoseconds to nanoseconds (see, for instance, [40]), and the association rates of the fastest binders, such as in the rigid barnase-barstar complex, are around 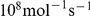. Thus is it reasonable to assume that conformational relaxation occurs significantly faster than the unbinding/binding process, from which it can be shown that 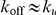. Thus, the rate of dissociation is approximately the rate of dissociation of the complexes in their bound conformational state, consistent with the above results. By contrast, the induced fit dissociation scheme can be modelled by the process
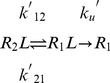
(7)From this, it can be shown that
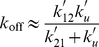
(8)It follows that
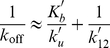
(9)Thus, from equations 9 and 2, the induced fit dissociation mechanism predicts the relationship
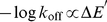
(10)where 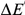 refers to the energy difference between the complex, 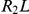 and the loosely bound 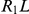, which implies that 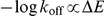. This relationship is not observed in the correlations between the terms and the dissociation rates; correlations with DFIRE_EBU and DDFIRE_EBU are 0.005 and −0.031 respectively. It could be that the contribution of 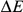 is small and only becomes apparent once the stronger interfacial energetics are factored out. However, combinations of interfacial and conformational energy terms were evaluated during feature selection and, with the exception of model **d**, were not selected as they did not provide better predictive value than when conformational energy terms are omitted. Thus, a key prediction of induced fit dissociation is not observed within the correlations, and is only borne out by one of the four best performing models. Finally, most of the terms in the 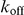 models are coarse-grain interaction energy terms. Although the rate of dissociation is clearly related to the specific atomic interactions at the interface, the selection of coarse-grain models over atomic potentials suggests that the rates of dissociation are best determined by evaluating low resolution recognition factors [41]. However, as these terms do not correlate significantly with the dissociation rates (p<0.01), it could be that the high resolution factors are at play, but not sufficiently modelled by the terms in the descriptor set.

### Summary and Conclusion

In this work, a set of empirical rate constants were derived from the literature and compared to a large set of molecular descriptors in order to find correlations with physical and energetic properties. While no highly significant correlations could be found with 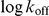, a number of correlations with 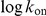 were identified. The most highly correlated factor found for the association rate is the energy difference between the unbound and bound conformational states. This signal can be detected by a number of different potentials, including coarse and atomistic pair potentials (DFIRE_EBU, OPUS_PSP_EBU, OPUS_CA_EBU, DDFIRE_EBU) and a potential that models the energetics of restructuring the intramolecular hydrogen bonding network (ROS_HBOND_UB). The signal is the strongest and most frequently found when averaged over ensembles of structures generated around the bound and unbound crystal structures. The second greatest factor suggested by the data is the role of intermolecular hydrogen bonding (HBOND_ENS and NUM_HB), suggesting an important role for water mediated intermediates along the binding pathway. When the empirical rate constants are filtered, so as to only include values that can be combined to produce binding affinities which are corroborated by further experiments, both the correlations and the number of significant correlations increase. These additional terms include other intermolecular hydrogen bonding term (ROS_HBOND, ROS_HBOND_ENS and H_BOND), three coarse-grained interaction pair potential energies (QP_PP, MJPL_PP and RO_PP), a side-chain entropy change term (STC_S_SC_ENS), London Dispersion terns (ROS_FA_ATR and ROS_FA_ATR_ENS) and continuum electrostatics energy changes (SASA, LK_SOLV and LK_SOLV_ENS).

Feature selection was then used to train a series of 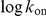 and 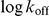 models using the descriptors. Each 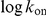 and 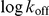 pair was then combined to predict the affinities of a separate set of complexes for which affinities are available, which was then used to select a pair of rate constant models for evaluation on another separate test set. A number of data partitioning and model selection schemes were evaluated, three of which were capable of reproducing the binding affinity of the final validation set with a correlation comparable to two state of the art potentials of mean force, and with lower RMSE. The features selected by these models strongly implicate hydrogen bonding as an important factor for efficient protein association, and suggest that low resolution recognition factors play a role in dissociation. However, the most significant conclusion of this study regards the role of conformational change. The mechanism through which proteins bind to one another has been a question of much debate. Structural studies have shown that the unbound proteins sample conformations close to the bound [18], [20], and theoretical work has identified the conditions under which the conformational selection mechanism is dominant [21], [42]. While the prominence of interactions with some excited state has been inferred from kinetic data in a small number of antibody/antigen systems [43]–[46], the correspondence to a state that is pre-organized for binding has not previously been shown. In this study we quantitatively demonstrate, using models which are automatically generated by machine learning with no *a priori* assumptions about binding mechanism, the distinctive association and dissociation kinetics which exemplify the conformational selection mechanism. Most significantly, the rate of association is linearly proportional to the pre-equilibrium constant, 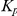, between the unbound and the bound conformational ensembles. Although the induced fit mechanism cannot be conclusively ruled out for all the cases considered here, only limited evidence could be found in support of it, suggesting that that it is too infrequent or its influence too subtle, to be discernible through the imprecisions inherent in the empirical data and theoretical models employed. These observations are shown using a functionally diverse set of complexes which undergo a large range of conformational changes upon binding and span several orders of magnitude in binding affinity. Consequently, they suggest a number of general strategies which could be employed for the engineering of rate constants. Specifically, the rate of association could be enhanced by introducing a mutation which preferentially stabilizes the internal energy of the bound conformational ensemble, or destabilized the unbound. Further, the role of hydrogen bonding suggests that one could modulate interaction turnover. Should it be possible to interconvert between intermolecular hydrogen bonds and other interactions, such as hydrophobic contacts, without disrupting affinity, then constructing an interface rich with hydrogen bonds would result in high association and dissociation rates, whilst an interface bereft of hydrogen bonds would have slower turnover. The methods presented here can provide estimates of the extent of these effects, and can be easily calculated. For instance, models **a** and **b** require only four descriptors, all of which can be determined using software and servers which are free and publicly available for academic use. The applied method has shown the utility of the three-state conformational selection kinetic model. This immediately suggests possible refinements to association rate models. Assuming conformational selection, 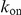 can be decomposed into 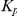 and 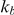 factors, of which the former can be modeled using equation 2 with the DFIRE_EBU descriptor, and the latter using one of previous methods developed in the rigid-body regime [10], [11]. Alternatively, 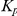 can be factored out of the empirical 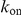 values, and the presented data mining technique can be applied for the prediction of 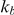. Additionally, the method presented here can be applied to the construction of protein binding thermodynamics models. For instance, feature selection can be used to construct 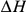 and 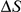 functions, which can be similarly selected and validated by being combined using the equation 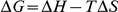 to predict binding free energies.

## Methods

The complexes and molecular descriptors used are as described in Moal et al. [13]. As the data is based on the structural affinity benchmark [12], pairs of complexes that are homologous at the family level are excluded from the data set, with the exception of cognate/non-cognate pairs for which one interaction has much lower affinity than the other. Thus, potential biases originating from predictions for complexes for which similar interactions appears in the training set, are unlikely to exaggerate the predictive value as determined by the validation set.

### Feature and Model Selection

**Figure 4:**
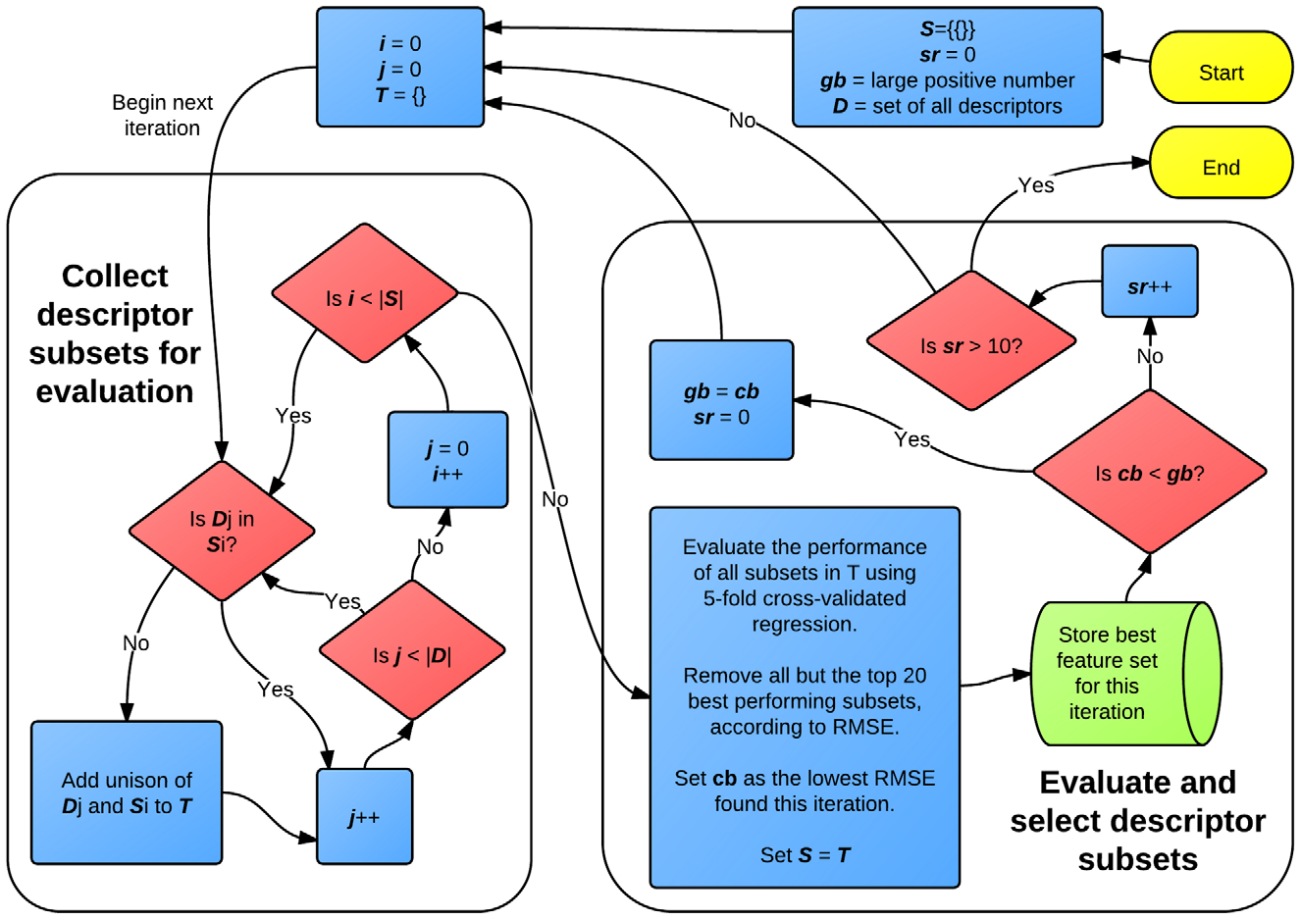
A Flowchart of the feature selection algorithm. The algorithm can be divided into two parts. In the first, a set of descriptor subsets, **T**, is constructed by first iterating over the set of descriptors subsets kept in the previous iteration, **S**. In the first iteration, **S** contains only the empty set. For each member, **S** 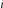, new descriptor subsets are created by combining **S** 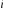 with each descriptor not already in **S** 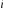. These are collected into **T**, and evaluated by their 5-fold cross-validated RMSE in the second part of the algorithm. The 20 best performing subsets are kept for the next iteration, and that with the lowest RMSE is stored for later model selection and validation. If the lowest RMSE in the current iteration, ***cb***, is higher than the lowest RMSE found in all previous iterations, ***gb***, then the speculative round counter, ***sr***, is incremented. Otherwise it is reset to 0. The algorithm terminates after 10 consecutive speculative rounds.

The feature selection and model building algorithm used is a population based algorithm with a population of 20; upon each iteration, 20 feature subsets are carried on to the next iteration. It is a forward selection algorithm in which the feature set grows by one feature per iteration. Further, it is a greedy algorithm, so that the 20 feature subsets which are carried onto the next iteration are those which give the greatest performance when evaluated. Performance is evaluated as the RMSE using linear regression and 5-fold cross-validation. On the first iteration of the algorithm, each molecular descriptor is evaluated on its own. The top 20 highest performing features are then retained as the feature subsets for the next iteration. In the second and all subsequent iterations, each of the previously retained feature subsets is evaluated in combination with every feature not in that subset. Again the top 20 subsets tested are retained for the next iteration. The algorithm proceeds up to 10 speculative rounds; should the cross-validated RMSE not decrease for 10 consecutive round, the algorithm terminates. At each iteration, a linear model is constructed by regression against the training data using the best performing descriptor subset. A flowchart outlining the feature selection scheme is shown in Figure 4. An early stopping surface is created by combining the series of 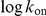 model and 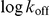 models using equation 1, which is then evaluated on the model selection test complexes. The pair of selected models is then combined to predict the affinities of the validation set. All parameters were chosen so as to give reasonable coverage of subset space, yet remain feasible. Parameters were not subsequently altered or optimized, so as to avoid possible biases arising from tinkering until the desired result is obtained. As it is the ratio of the predicted rate constants which is used for model selection and validation, it is possible that this scheme could systematically overestimate or underestimate 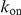, provided that the 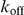 model is also systematically biased in a compensatory way so as to generate accurate binding free energies, and *vice versa*. However, as the models which are being selected are trained on empirical rate constants, and the number of pairs of rate functions of combined predictive value is small, it is unlikely that such a pair of models would be generated and selected, and thus this potential source of bias is negligible.

## Supporting Information

Dataset S1 Values and descriptors for log10 *k*_on_.
(CSV)

Dataset S2 Values and descriptors for log10 *k*_off_.
(CSV)

Table S1 Empirical kinetic rates constants extracted from the literature.
(PDF)

Table S2 Constructed log10 *k*_on_ and log10 *k*_off_ models.
(PDF)
