# Supplementary material for: Kinetic Rate Constant Prediction Supports the Conformational Selection Mechanism of Protein Binding
Source: PLoS Comput Biol. 2012 Jan 12;8(1):e1002351. doi: 10.1371/journal.pcbi.1002351 (PMC3257286; doi:10.1371/journal.pcbi.1002351)
Supplement: Table S1 — Empirical kinetic rates constants extracted from the literature. (PDF) [file pcbi.1002351.s003.pdf]

Table 1: The rate constant benchmark. Complexes overlapping with the validated set are higlighted in **bold**. The first entry refers to pdb code for the bound complex. I-RMSD values were calculated using C_α positions. †Personal communication

Complex	Type	Protein 1	PDB1	Protein 2	PDB2	k_{on} ($mol^{-1}s^{-1}$)	k_{off} (s^{-1})	DASA (Å^2)	I-RMSD (Å)	Ref.
1CBW_ABC:D	EI	Chymotrypsin	4CHA_ABC	BPTI	9PTI_A	1.7e5	1.8e-3	1457	0.74	(1)
**1DFJ_E:I**	EI	Ribonuclease A	9RSA_B	Rnase inhibitor	2BNH_A	1.66e8	9.8-6	2582	1.02	(2)
**1E4K_AB:C**	OR	FC fragment of human IgG 1	2DTQ_AB	Human FCGR III	1FNL_A	4.2e5	7.5e-1	1634	2.59	(3)
1E6E_A:B	ES	Adrenoxin reductase	1E1N_A	Adrenoxin	1CJE_D	4.434e3	3.8e-3	2315	1.33	(4)
1E6J_HL:P	A	Fab 13B5	1E6O_HL	HIV-1 capsid protein p24	1A43_A	3.5e5	1.2e-3	1245	1.05	(5)
**1EER_A:BC**	OR	Erythropoietin	1BUY_A	EPO receptor	1ERN_AB	2.1e7	7.8e-5	3347	2.44	(6)
1EMV_A:B	EI	Colicin E9 nuclease	1FSJ_B	Im9 immunity protein	1IMQ_A	9.0e7	2.2e-6	1535	1.28	(7)
**1FFW_A:B**	OX	Chemotaxis protein CheY	3CHY_A	Chemotaxis protein CheA	1FWP_A	3.68e2	1.14e-5	1170	1.43	(8)
1FSK_BC:A	AB	Fab - Birch pollen antigen Bet V1	1FSK_BC	Birch pollen antigen Bet V1	1BV1_A	1.0e5	2.5e-5	1623	0.45	†
**1GL1_A:I**	EI	Chymotrypsin	4CHA_ABC	PMP-C (LCMI II)	1PMC_A(6)	8.00e5	1.62e-4	1595	1.2	(9)
**1GXD_A:C**	EI	ProMMP2 type IV collagenase	1CK7_A	Metalloproteinase inhibitor 2	1BR9_A	1.4e5	7e-4	2445	1.39	(10)
1JIW_P:I	EI	Alkaline metallo-proteinase	1AKL_A	Proteinase inhibitor	2RN4_A(1)	2.5e5	1.15e-6	2018	2.07	(11)
1JMO_A:HL	ER	Heparin cofactor	1JMJ_A	Thrombin	2CN0_HL	2.23e2	2.5e-5	3461	3.21	(12)
1JPS_HL:T	A	Fab D3H44	1JPT_HL	Tissue factor	1TFH_B	9.8e5	1e-4	1852	0.51	(13)
**1JTG_B:A**	EI	β-lactamase inhibitor protein	3GMU_B	β-lactamase TEM-1	1ZG4_A	3.1e5	1.2e-4	2600	0.49	(14)
**1JWH_CD:A**	ER	Casein kinase II β chain	3EED_AB	Casein kinase II α chain	3C13_A	6.65e4	3.6e-4	1451	1.27	(15)
**1KAC_A:B**	OR	Adenovirus fiber knob protein	1NOB_F	Adenovirus receptor	1F5W_B	1.8e5	2.8e-3	1456	0.95	(16)
1KKL_ABC:H	ES	HPr kinase C-ter domain	1JB1_ABC	HPr	2HPR_A	1.3e5	5.8e-3	1641	2.2	(17)
1KLU_AB:D	OX	MHC class 2 HLA-DR1	1H15_AB	Staphylococcus enterotoxin C3	1STE_A	5.22e3	2.4e-2	1254	0.43	(18)
**1KTZ_A:B**	OR	TGF-β	1TGK_A	TGF-β receptor	1M9Z_A	7.4e5	5.4e-2	989	0.39	(19)
1KXQ_H:A	AB	Camel VHH - Pancreatic α-amylase	1KXQ_H	Pancreatic α-amylase	1PPI_A	2.36e5	8e-4	2172	0.72	(20)
**1LFD_B:A**	OG	Ras.GNP	5P21_A	RalGDS Ras-interacting domain	1LXD_A	7.7e6	1.49e1	1167	1.79	(21)
**1MAH_A:F**	EI	Acetylcholinesterase	1J06_B	Fasciculin	1FSC_A	2.7e7	2.9e-5	2145	0.61	(22)
**1MLC_AB:E**	A	Fab44.1	1MLB_AB	HEW lysozyme	3LZT_A	1e4	9.1e-4	1392	0.6	(23)
**1MQ8_A:B**	OX	ICAM-1 domain 1-2	1IAM_A	Integrin α-L I domain	1MQ9_A	1.33e5	4.3e-1	1241	1.76	(24)
1P2C_AB:C	A	FabF10.6.6	2Q76_AB	HEW lysozyme	3LZT_A	4.21e4	2.92e-3	1456	0.46	(25)
**1PPE_E:I**	EI	Trypsinogen	2PTN_A	CMTI-1 squash inhibitor	1LU0_A	6.7e6	2.5e-5	1688	0.34	(26)
**1T6B_X:Y**	OR	Anthrax protective antigen	1ACC_A	Anthrax toxin receptor	1SHU_X	5.3e4	9.2e-6	1948	0.62	(27)
**1VFB_AB:C**	A	Fv D1.3	1VFA_AB	HEW lysozyme	8LYZ_A	1.39e6	5.14e-3	1383	1.02	(28)
**1XU1_ABD:T**	OR	TNF domain of APRIL	1U5Y_ABD	TACI CRD2 domain	1XUT_A(11)	9.22e5	5.89e-3	1700	1.3	(29)
2AJF_A:E	OR	Angiotensin-converting enzyme 2	1R42_A	SARS spike protein receptor binding domain	2GHV_E	7.12e4	1.16e-3	1704	0.65	(30)
2B42_A:B	EI	Xylanase	2DCY_A	Xylanase inhibitor	1T6E_X	3.61e5	3.6e-4	2520	0.72	(31)
**2B4J_AB:C**	OX	Integrase (HIV-1)	1BIZ_AB	PC4 and SFRS1 interacting protein	1Z9E_A(1)	4.75e5	3.9e-3	1259	0.99	(32)
**2GOX_A:B**	OX	Complement C3d fragment	1C3D_A	Staphylococcus aureus Efb-C	2GOM_A	4.04e5	5.63e-4	1631	0.6	(33)
2I25_N:L	A	Shark single domain antigen receptor	2I24_N	HEW lysozyme	3LZT_A	9e4	1e-4	1425	1.21	(34)
**2I9B_E:A**	OR	uPAR surface receptor	1YWH_A	Urokinase-type plasminogen activator	2I9A_A	1.19e5	1.14e-4	2382	3.79	(35)
**2OZA_B:A**	OX	MAP kinase 14	3HEC_A	MAP kinase-activated protein kinase 2	3FYK_X	8.1e7	8e-2	6254	1.89	(36)
**2PTC_E:I**	EI	Trypsinogen	2PTN_A	BPTI	9PTI_A	1.1e6	6.6e-8	1429	0.28	(37)
**2SIC_E:I**	EI	Subtilisin	1SUP_A	Streptomyces subtilisin inhibitor	3SSI_A	6.5e6	9e-5	1617	0.36	(38)
**2SNI_E:I**	EI	Subtilisin	1UBN_A	Chymotrypsin inhibitor 2	2CI2_I	3.1e6	6.1e-6	1628	0.35	(39)
2VIR_AB:C	A	Fab	1GIG_LH	Flu virus hemagglutinin	2HMG_AB	1.1e5	1.1e-4	1263	0.8	(40)
2VIS_AB:C	A	Fab	1GIG_LH	Flu virus hemagglutinin	2VIU_ACE	5.4e2	2.16e-3	1296	0.8	(40)
**2WPT_A:B**	EI	Colicin E9 nuclease	1FSJ_B	Im2 immunity protein	2NO8_A	5e7	7.3e-1	1581	1.61	(41)
3BP8_AB:C	OX	Mlc transcription regulator	1Z6R_AB	PTS glucose-specific enzyme EIICB	3BP3_A	9.95e5	3.85e-3	1398	0.45	(42)

References

1. Castro MJ, Anderson S. Alanine point-mutations in the reactive region of bovine pancreatic trypsin inhibitor: effects on the kinetics and thermodynamics of binding to beta-trypsin and alpha-chymotrypsin. Biochemistry 1996;35:11 435–11 446.

2. Vicentini AM, Kieffer B, Matthies R, Meyhack B, Hemmings BA, Stone SR, Hofsteenge J. Protein chemical and kinetic characterization of recombinant porcine ribonuclease inhibitor expressed in Saccharomyces cerevisiae. Biochemistry 1990;29:8827–8834.

3. Maenaka K, van der Merwe PA, Stuart DI, Jones EY, Sondermann P. The human low affinity Fcgamma receptors IIa, IIb, and III bind IgG with fast kinetics and distinct thermodynamic properties. J Biol Chem 2001;276:44 898–44 904.

4. Schiffler B, Zollner A, Bernhardt R. Stripping down the mitochondrial cholesterol hydroxylase system, a kinetics study. J Biol Chem 2004;279:34 269–34 276.

5. Monaco-Malbet S, Berthet-Colominas C, Novelli A, Battai N, Piga N, Cheynet V, Mallet F, Cusack S. Mutual conformational adaptations in antigen and antibody upon complex formation between an Fab and HIV-1 capsid protein p24. Structure 2000;8:1069–1077.

6. Darling RJ, Kuchibhotla U, Glaesner W, Micanovic R, Witcher DR, Beals JM. Glycosylation of erythropoietin affects receptor binding kinetics: role of electrostatic interactions. Biochemistry 2002;41:14 524–14 531.

7. Wallis R, Leung KY, Pommer AJ, Videler H, Moore GR, James R, Kleanthous C. Protein-protein interactions in colicin E9 DNase-immunity protein complexes. 2. Cognate and noncognate interactions that span the millimolar to femtomolar affinity range. Biochemistry 1995;34:13 751–13 759.

8. Schuster SC, Swanson RV, Alex LA, Bourret RB, Simon MI. Assembly and function of a quaternary signal transduction complex monitored by surface plasmon resonance. Nature 1993;365:343–347.

9. Kellenberger C, Boudier C, Bermudez I, Bieth JG, Luu B, Hietter H. Serine protease inhibition by insect peptides containing a cysteine knot and a triple-stranded beta-sheet. J Biol Chem 1995;270:25 514–25 519.

10. Olson MW, Gervasi DC, Mobashery S, Fridman R. Kinetic analysis of the binding of human matrix metalloproteinase-2 and -9 to tissue inhibitor of metalloproteinase (TIMP)-1 and TIMP-2. J Biol Chem 1997;272:29 975–29 983.

11. Feltzer RE, Gray RD, Dean WL, Pierce WM. Alkaline proteinase inhibitor of Pseudomonas aeruginosa. Interaction of native and N-terminally truncated inhibitor proteins with Pseudomonas metalloproteinases. J Biol Chem 2000;275:21 002–21 009.

12. Ciaccia AV, Monroe DM, Church FC. Arginine 200 of heparin cofactor II promotes intramolecular interactions of the acidic domain. Implication for thrombin inhibition. J Biol Chem 1997;272:14 074–14 079.

13. Presta L, Sims P, Meng YG, Moran P, Bullens S, Bunting S, Schoenfeld J, Lowe D, Lai J, Rancatore P, *et al.*. Generation of a humanized, high affinity anti-tissue factor antibody for use as a novel antithrombotic therapeutic. Thromb Haemost 2001;85:379–389.

14. Albeck S, Schreiber G. Biophysical characterization of the interaction of the beta-lactamase TEM-1 with its protein inhibitor BLIP. Biochemistry 1999;38:11–21.

15. Martel V, Filhol O, Nueda A, Cochet C. Dynamic localization/association of protein kinase CK2 subunits in living cells: a role in its cellular regulation? Ann N Y Acad Sci 2002;973:272–277.

16. Kirby I, Davison E, Beavil AJ, Soh CP, Wickham TJ, Roelvink PW, Kovesdi I, Sutton BJ, Santis G. Identification of contact residues and definition of the CAR-binding site of adenovirus type 5 fiber protein. J Virol 2000;74:2804–2813.

17. Lavergne JP, Jault JM, Galinier A. Insights into the functioning of Bacillus subtilis HPr kinase/phosphatase: affinity for its protein substrates and role of cations and phosphate. Biochemistry 2002;41:6218–6225.

18. Andersen PS, Lavoie PM, Sekaly RP, Churchill H, Kranz DM, Schlievert PM, Karjalainen K, Mariuzza RA. Role of the T cell receptor alpha chain in stabilizing TCR-superantigen-MHC class II complexes. Immunity 1999;10:473–483.

19. Baardsnes J, Hinck CS, Hinck AP, O'Connor-McCourt MD. TbetaR-II discriminates the high- and low-affinity TGF-beta isoforms via two hydrogen-bonded ion pairs. Biochemistry 2009;48:2146–2155.

20. Lauwereys M, Arbabi Ghahroudi M, Desmyter A, Kinne J, Holzer W, De Genst E, Wyns L, Muyldermans S. Potent enzyme inhibitors derived from dromedary heavy-chain antibodies. EMBO J 1998;17:3512–3520.

21. Kiel C, Selzer T, Shaul Y, Schreiber G, Herrmann C. Electrostatically optimized Ras-binding Ral guanine dissociation stimulator mutants increase the rate of association by stabilizing the encounter complex. Proc Natl Acad Sci USA 2004;101:9223–9228.

22. Eastman J, Wilson EJ, Cervenansky C, Rosenberry TL. Fasciculin 2 binds to the peripheral site on acetylcholinesterase and inhibits substrate hydrolysis by slowing a step involving proton transfer during enzyme acylation. J Biol Chem 1995;270:19 694–19 701.

23. Goldbaum FA, Cauerhff A, Velikovsky CA, Llera AS, Riottot MM, Poljak RJ. Lack of significant differences in association rates and affinities of antibodies from short-term and long-term responses to hen egg lysozyme. J Immunol 1999;162:6040–6045.

24. Shimaoka M, Xiao T, Liu JH, Yang Y, Dong Y, Jun CD, McCormack A, Zhang R, Joachimiak A, Takagi J, *et al.*. Structures of the alpha L I domain and its complex with ICAM-1 reveal a shape-shifting pathway for integrin regulation. Cell 2003;112:99–111.

25. Cauerhff A, Goldbaum FA, Braden BC. Structural mechanism for affinity maturation of an anti-lysozyme antibody. Proc Natl Acad Sci USA 2004;101:3539–3544.

26. Otlewski J, Zbyryt T. Single peptide bond hydrolysis/resynthesis in squash inhibitors of serine proteinases. 1. Kinetics and thermodynamics of the interaction between squash inhibitors and bovine beta-trypsin. Biochemistry 1994;33:200–207.

27. Wigelsworth DJ, Krantz BA, Christensen KA, Lacy DB, Juris SJ, Collier RJ. Binding stoichiometry and kinetics of the interaction of a human anthrax toxin receptor, CMG2, with protective antigen. J Biol Chem 2004;279:23 349–23 356.

28. Foote J, Winter G. Antibody framework residues affecting the conformation of the hypervariable loops. J Mol Biol 1992;224:487–499.

29. Wu Y, Bressette D, Carrell JA, Kaufman T, Feng P, Taylor K, Gan Y, Cho YH, Garcia AD, Gollatz E, *et al.*. Tumor necrosis factor (TNF) receptor superfamily member TACI is a high affinity receptor for TNF family members APRIL and BLyS. J Biol Chem 2000;275:35 478–35 485.

30. Li W, Zhang C, Sui J, Kuhn JH, Moore MJ, Luo S, Wong SK, Huang IC, Xu K, Vasilieva N, *et al.*. Receptor and viral determinants of SARS-coronavirus adaptation to human ACE2. EMBO J 2005;24:1634–1643.

31. Fierens K, Gils A, Sansen S, Brijs K, Courtin CM, Declerck PJ, De Ranter CJ, Gebruers K, Rabijns A, Robben J, *et al.*. His374 of wheat endoxylanase inhibitor TAXI-I stabilizes complex formation with glycoside hydrolase family 11 endoxylanases. FEBS J 2005;272:5872–5882.

32. Tsiang M, Jones GS, Hung M, Mukund S, Han B, Liu X, Babaoglu K, Lansdon E, Chen X, Todd J, *et al.*. Affinities between the binding partners of the HIV-1 integrase dimer-lens epithelium-derived growth factor (IN dimer-LEDGF) complex. J Biol Chem 2009;284:33 580–33 599.

33. Haspel N, Ricklin D, Geisbrecht BV, Kavraki LE, Lambris JD. Electrostatic contributions drive the interaction between Staphylococcus aureus protein Efb-C and its complement target C3d. Protein Sci 2008;17:1894–1906.

34. Dooley H, Stanfield RL, Brady RA, Flajnik MF. First molecular and biochemical analysis of in vivo affinity maturation in an ectothermic vertebrate. Proc Natl Acad Sci USA 2006;103:1846–1851.

35. Gardsvoll H, Werner F, S?ndergaard L, Dan? K, Ploug M. Characterization of low-glycosylated forms of soluble human urokinase receptor expressed in Drosophila Schneider 2 cells after deletion of glycosylation-sites. Protein Expr Purif 2004;34:284–295.

36. Lukas SM, Kroe RR, Wildeson J, Peet GW, Frego L, Davidson W, Ingraham RH, Pargellis CA, Labadia ME, Werneburg BG. Catalysis and function of the p38 alpha.MK2a signaling complex. Biochemistry 2004;43:9950–9960.

37. Olson ST, Bock PE, Kvassman J, Shore JD, Lawrence DA, Ginsburg D, Bjork I. Role of the catalytic serine in the interactions of serine proteinases with protein inhibitors of the serpin family. Contribution of a covalent interaction to the binding energy of serpin-proteinase complexes. J Biol Chem 1995;270:30 007–30 017.

38. Masuda-Momma K, Hatanaka T, Inouye K, Kanaori K, Tamura A, Akasaka K, Kojima S, Kumagai I, Miura K, Tonomura B. Interaction of subtilisin BPN’ and recombinant Streptomyces subtilisin inhibitors with substituted P1 site residues. J Biochem 1993;114:553–559.

39. Otzen DE, Fersht AR. Analysis of protein-protein interactions by mutagenesis: direct versus indirect effects. Protein Eng 1999;12:41–45.

40. Fleury D, Wharton SA, Skehel JJ, Knossow M, Bizebard T. Antigen distortion allows influenza virus to escape neutralization. Nat Struct Biol 1998;5:119–123.

41. Li W, Hamill SJ, Hemmings AM, Moore GR, James R, Kleanthous C. Dual recognition and the role of specificity-determining residues in colicin E9 DNase-immunity protein interactions. Biochemistry 1998;37:11 771–11 779.

42. Nam TW, Jung HI, An YJ, Park YH, Lee SH, Seok YJ, Cha SS. Analyses of Mlc-IIBGlc interaction and a plausible molecular mechanism of Mlc inactivation by membrane sequestration. Proc Natl Acad Sci USA 2008;105:3751–3756.
