# Supplementary material for: Kinetic Rate Constant Prediction Supports the Conformational Selection Mechanism of Protein Binding
Source: PLoS Comput Biol. 2012 Jan 12;8(1):e1002351. doi: 10.1371/journal.pcbi.1002351 (PMC3257286; doi:10.1371/journal.pcbi.1002351)
Supplement: Table S2 — Constructed log10 k on and log10 k off models. (PDF) [file pcbi.1002351.s004.pdf]

The following tables show the rate constant regression models generated using the four schemes for partitioning the data and the two criteria used for model selection. For each feature, absolute weights (W) and normalised weights (W_n), found after converting to z-scores, are shown. The term CONSTANT refers to the constant determined during regression.

Table 1: Rate constant models

		$\log_{10} k_{on}$			$\log_{10} k_{off}$			ΔG_{sel}	ΔG_{val}	
Sch.	Sel.	Feat.	W	W_n	Feat.	W	W_n	Corr.	Corr.	p
1	RMSE	CONSTANT	4.29	-	CONSTANT	-1.40	-	0.69	0.09	0.45
		NUM_HB	0.07	0.52	ROS_CG_BETA	-0.79	-0.85			
		DFIRE_EBU	-0.00	-0.50	TES_PP	0.10	0.35			
					ROS_FA_REP_UB	0.05	0.35			
					ACE19_HYDR_UB	0.00	0.43			
					OPUS_CA_ENS	0.03	0.57			
2	RMSE	CONSTANT	4.29	-	CONSTANT	-2.11	-	0.10	0.59	<0.01
		NUM_HB	0.07	0.52	ROS_CG_BETA	-0.68	-0.73			
		DFIRE_EBU	-0.00	-0.50	OPUS_CA_ENS	0.04	0.67			
3	RMSE	CONSTANT	6.13	-	CONSTANT	-6.32	-	0.60	0.19	0.14
		RES_C	-0.07	-0.51	ROS_CG_BETA	-0.49	-0.52			
		NUM_HB	0.08	0.44	NIP	8612.18	0.51			
		ACE22_ALL	0.00	0.21						
		ROS_FA_REP	0.04	0.23						
		ROS_CG_VDW	-0.76	-0.19						
		STC_H	-0.05	-0.24						
		GEN_4_BODY_UB	0.01	0.40						
		DFIRE_EBU	-0.00	-0.47						
4	RMSE	CONSTANT	4.18	-	CONSTANT	-6.32	-	0.17	0.51	<0.01
		NUM_HB	0.07	0.39	ROS_CG_BETA	-0.49	-0.52			
		DFIRE_EBU	-0.00	-0.47	NIP	8612.18	0.51			
1	Corr.	CONSTANT	4.29	-	CONSTANT	-5.51	-	0.69	0.12	0.29
		NUM_HB	0.07	0.52	ROS_CG_BETA	-0.78	-0.85			
		DFIRE_EBU	-0.00	-0.50	NIP	8744.08	0.50			
					OPUS_CA	-0.03	-0.59			
					TES_PP	0.13	0.46			
					MJPL_PP_UB	0.00	0.28			
					OPUS_CA_ENS	0.05	0.85			
2	Corr.	CONSTANT	4.84	-	CONSTANT	-1.32	-	0.22	0.39	0.03
		NUM_HB	0.09	0.61	ROS_CG_BETA	-0.77	-0.82			
		BIOSIMZ_KON	-0.02	-0.37	TES_PP	0.10	0.35			
		ROS_HBOND_UB	-0.04	-0.34	ACE19_HYDR_UB	0.00	0.30			
		TES_PP_UB	0.02	0.52	OPUS_CA_ENS	0.04	0.65			
		DFIRE_EBU	-0.00	-0.49						
3	Corr.	CONSTANT	5.17	-	CONSTANT	-2.65	-	0.72	0.03	0.85
		RES_C	-0.04	-0.32	PLANARITY	-1.11	-0.80			
		NUM_HB	0.08	0.44	ECCENTRIC	-1.64	-0.14			
		DFIRE_EBU	-0.00	-0.47	NUM_HB	0.03	0.14			
					ACE22_ALL	0.00	0.20			
					ROS_CG_BETA	-1.00	-1.07			
					MSBM_PP	-2.11	-0.56			
					ROS_CG_BETA_UB	0.41	0.51			
					ROS_HBOND_UB	0.09	0.62			
					DFIRE_UB	-0.09	-0.43			
					RF_PP_UB	0.04	0.53			
					QP_PP_UB	0.03	0.46			
					SKOB_PP_UB	-0.07	-0.98			
					MJPL_PP_UB	-0.00	-0.20			
					TD_PP_UB	0.09	0.95			

Models continued on next page

		$\log_{10} k_{\text{on}}$			$\log_{10} k_{\text{off}}$			ΔG_{sel}	ΔG_{val}	
Sch.	Sel.	Feat.	W	W_n	Feat.	W	W_n	Corr.	Corr.	p
					RO_PP_UB	-0.01	-0.33			
					ROS_FA_REP_ENS	0.06	0.43			
					ROS_HBOND_ENS	-0.35	-0.27			
					S_WLC_ALL2	-0.36	-0.72			
4	Corr.	CONSTANT	6.40	-	CONSTANT	-6.32	-	0.27	0.43	0.02
		RES_C	-0.07	-0.49	ROS_CG_BETA	-0.49	-0.52			
		NUM_HB	0.07	0.39	NIP	8612.18	0.51			
		ACE22_ALL	0.00	0.28						
		ROS_FA_REP	0.05	0.27						
		ROS_CG_VDW	-0.77	-0.20						
		STC_H	-0.06	-0.26						
		ROS_TOTAL_UB	0.01	0.13						
		GEN_4_BODY_UB	0.02	0.41						
		ROS_FA_PP_EBU	0.59	0.23						
		DFIRE_EBU	-0.00	-0.54						

Table 2: Rate constant models (2OZA omitted)

		$\log_{10} k_{\text{on}}$			$\log_{10} k_{\text{off}}$			ΔG_{sel}	ΔG_{val}	
Sch.	Sel.	Feat.	W	W_n	Feat.	W	W_n	Corr.	Corr.	p
1	RMSE	CONSTANT	4.81	-	CONSTANT	1.02	-	0.51	0.08	0.48
		OPUS_PSP_EBU	-0.00	-0.56	CATION_PI	0.59	0.49			
					TD_PP	0.31	0.59			
					GEOMETRIC_EBU	0.05	0.71			
					RF_PP_EBU	-0.20	-0.43			
2	RMSE	CONSTANT	4.81	-	CONSTANT	-0.45	-	0.00	0.48	$<$0.01
		OPUS_PSP_EBU	-0.00	-0.56	TD_PP	0.28	0.52			
					ROS_CG_VDW_ENS	-4.55	-0.43			
3	RMSE	CONSTANT	4.44	-	CONSTANT	-0.45	-	0.72	0.25	0.05
		NUM_HB	0.07	0.37	ROS_CG_BETA	-0.45	-0.35			
		ROS_CG_BETA	0.45	0.44	MJ2H_PP	0.02	0.73			
		GEOMETRIC	-0.01	-0.30	SKOB_PP_UB	-0.05	-0.82			
		ROS_HBOND_UB	-0.12	-1.09	MJPL_PP_UB	0.00	0.15			
		DDFIRE_UB	0.06	0.96	TD_PP_UB	0.08	0.83			
		GEN_4_BODY_UB	0.01	0.33						
		RO_PP_UB	-0.00	-0.31						
		ROS_CG_VDW_ENS	-1.57	-0.23						
		DFIRE_EBU	-0.00	-0.28						
4	RMSE	CONSTANT	5.80	-	CONSTANT	-0.87	-	0.32	0.59	$<$0.01
		RES_C	-0.07	-0.53	MJ2H_PP	0.01	0.46			
		NUM_HB	0.08	0.42						
		ROS_CG_VDW	-1.01	-0.27						
		STC_H	-0.06	-0.28						
		GEN_4_BODY_UB	0.01	0.39						
		DFIRE_EBU	-0.00	-0.41						
		S_WLC_INT2	-0.28	-0.19						
1	Corr.	CONSTANT	4.81	-	CONSTANT	-0.50	-	0.52	0.09	0.43
		OPUS_PSP_EBU	-0.00	-0.56	RES_C	0.05	0.30			
					CATION_PI	0.59	0.49			
					TD_PP	0.27	0.52			
					GEOMETRIC_EBU	0.05	0.70			
					RF_PP_EBU	-0.16	-0.34			
2	Corr.	CONSTANT	4.30	-	CONSTANT	1.99	-	0.31	0.39	0.03
		NUM_HB	0.07	0.49	RES_NP	-0.03	-0.15			
		DFIRE_EBU	-0.00	-0.52	PLANARITY	0.02	0.01			
					NUM_SB	0.08	0.62			
					GAP_VOL	0.00	0.21			
					ROS_CG_VDW	-4.63	-0.42			

Models continued on next page

		$\log_{10} k_{\text{on}}$			$\log_{10} k_{\text{off}}$			ΔG_{sel}	ΔG_{val}	
Sch.	Sel.	Feat.	W	W_n	Feat.	W	W_n	Corr.	Corr.	p
					ROS_CG_ENV	-0.02	-0.13			
					ROS_HBOND	0.02	0.02			
					DELISI_SOLV	-0.05	-0.20			
					CATION_PI	0.45	0.38			
					ALIPHATIC	0.07	0.09			
					TD_PP	0.39	0.73			
					TES_PP	-0.01	-0.03			
					ROS_CG_ENV_UB	-0.05	-0.24			
					INTERNAL_UB	0.00	0.01			
					ACE19_HYDR_UB	0.01	0.52			
					ROS_FA_REP_EBU	-0.02	-0.10			
					ROS_FA_PP_EBU	-0.21	-0.06			
					GEOMETRIC_EBU	0.05	0.73			
					RF_PP_EBU	-0.21	-0.46			
					S_GP_ALL2	0.00	0.00			
					S_WLC_INT2	0.64	0.30			
3	Corr.	CONSTANT	4.44	-	CONSTANT	-0.45	-	0.72	0.25	0.05
		NUM_HB	0.07	0.37	ROS_CG_BETA	-0.45	-0.35			
		ROS_CG_BETA	0.45	0.44	MJ2H_PP	0.02	0.73			
		GEOMETRIC	-0.01	-0.30	SKOB_PP_UB	-0.05	-0.82			
		ROS_HBOND_UB	-0.12	-1.09	MJPL_PP_UB	0.00	0.15			
		DDFIRE_UB	0.06	0.96	TD_PP_UB	0.08	0.83			
		GEN_4_BODY_UB	0.01	0.33						
		RO_PP_UB	-0.00	-0.31						
		ROS_CG_VDW_ENS	-1.57	-0.23						
		DFIRE_EBU	-0.00	-0.28						
4	Corr.	CONSTANT	5.80	-	CONSTANT	-0.67	-	0.33	0.60	<0.01
		RES_C	-0.07	-0.53	MJ2H_PP	0.01	0.53			
		NUM_HB	0.08	0.42	MJPL_PP_UB	0.00	0.40			
		ROS_CG_VDW	-1.01	-0.27						
		STC_H	-0.06	-0.28						
		GEN_4_BODY_UB	0.01	0.39						
		DFIRE_EBU	-0.00	-0.41						
		S_WLC_INT2	-0.28	-0.19						
